# Digital (in)equalities and user emancipation: Examining the potential of Adorno's maxim of Mündigkeit for critical intergenerational learning

**DOI:** 10.3389/fsoc.2022.983034

**Published:** 2022-11-25

**Authors:** Miranda Leontowitsch, Friedrich Wolf, Frank Oswald

**Affiliations:** Department of Educational Sciences, Goethe University Frankfurt, Frankfurt, Germany

**Keywords:** post-digital age, artificial intelligence, algorithms, inequalities, learning, reflection, generations, emancipation toward autonomy

## Abstract

The widespread use of mobile technologies has penetrated the lives of people across all age groups with the usage of smartphones and wearables appearing “natural” and without alternatives. The digitalisation of everyday life means that communication and negotiation of social and societal meanings are co-constructed by users and mobile technologies thereby blurring the boundary between on- and off-line as well as social and private spheres. At the same time, the global-market logic that has driven the extent and speed of this social transformation raises questions as to how individuals retain influence and agency over the digital technologies that have come to define both social and private spheres and that surround them at all times. Against this backdrop, this theoretical paper discusses the role of Adorno's maxim of emancipation toward autonomy (Mündigkeit) for education (Erziehung) and critical learning *about* and living *with* digital technologies. The paper suggests a way forward through intergenerational learning as a didactical method of enhancing emancipation among younger and older generations of “users” in their joint efforts of becoming critical agents in an age of digitally enhanced data markets.

## Introduction

The widespread use of mobile technologies has penetrated the lives of people across all age groups with the usage of smartphones and tablets appearing “natural”. However, processes of digitalisation or digitisation and their impact on societal transformation into the digital age are more than the usage of digital technologies. The digitalisation of everyday life means that communication and negotiation of social and societal meanings are co-constructed by users and mobile technologies thereby blurring the boundary between on- and off-line (Jordan, 2009). The high degree of digitalisation in all age groups and the associated co-constitution of social processes through digital systems is a central argument why today's societies can be described as post-digital (Cramer, 2015). The term thus expresses the naturalization of digital systems and, at the same time, the impossibility of thinking of analog spaces as independent of digital ones. This perspective adds new cultural expectations of the social, togetherness and mutuality, and has the potential to perpetuate inequalities between social categories, particularly those of age, gender and education. As employment restructuring and opportunities have forced younger generations to leave their hometowns, digitalisation has contributed to new forms of connectedness of young and old generations within families (Amaro and Fonseca, 2013). However, the increase in the numbers of older people using mobile technologies continues to show divisions by gender, cultural background and income (Robinson et al., 2015) thus contributing to social inequalities.

Moreover, the post-digital age is characterized by classifying algorithms, so-called artificial intelligence. From online searches to navigation or browsing through one's own newsfeed, the results displayed there are based on the user's prior classification. These classifications, which are essential for the use of digital technologies, can be seen as hidden subjectifications, which are either perpetuated in the digital world or are newly created. Either way they decisively shape analog and digital spaces of action. Thus, critical voices have highlighted concerns regarding external control as well as data security in the everyday use of digital mobile technologies (Helbing et al., 2019). More generally, digitalisation has changed the meaning of things (i.e., smart phones) both in social relationships and in gaining access to relationships. These concerns are set against a backdrop of social transformations, with lifestyles and values becoming more pluralistic, social and organizational orders losing importance, and generational cohesion becoming more fragile. However, age groups are framed differently when it comes to problematising life in the post-digital age. Younger age groups are considered to need more protection from digital influences (Megele and Buzzi, 2017), whereas the use of digital technologies among older age groups is promoted as a way of enhancing social participation or independence for living at home (Schulz et al., 2015). Such views are built upon constructions of age and aging, and risk to polarize age and generational groups, and ignore the similarities all users of digital technologies face in terms of individual rights and autonomy. Indeed, both younger and older users lack role models for living in the post-digital age and both can have little understanding of the mechanisms behind the technologies they use. This raises questions as to what skills and knowledge young and old users need for life during rapid post-digital transformations.

Against this backdrop, this theoretical paper discusses the role of Adorno's maxim of emancipation toward autonomy (Mündigkeit) for education (Erziehung) and critical learning *about* and living *with* digital technologies. Adorno's critical social theory sets out to understand how individual and collective agency in modern capitalist societies is inextricably linked with unequal structures of power and wealth. Emancipation to autonomy has gained in acuteness through digitally enhanced globalization, which has enabled companies such as Alphabet, Amazon or Meta to become pan-national co-operations increasingly acting beyond national regulations at the same time as having direct influence on people's lives. Although this tech economy was not around at the time of Adorno's writing, the underlying economic systems of capitalism, that remain relevant to this day, are central to obscuring class differences of wealth and power behind a façade of leveled democracy or indeed digital liberalism.

Next to the theoretical analysis of emancipation in the digital age, the paper suggests a way forward through intergenerational learning as a didactical method of enhancing emancipation among different age groups of “users” in their joint efforts of becoming critical agents in an age of digitally enhanced economic domination. In doing so, the paper argues, that intergenerational learning that is concerned with difference and learning about otherness through reflection is well suited to allowing young and old people to find out what role digital technologies play in their lives, how much agency they as users have, and how they envisage their future in the post-digital age.

## Digital (in)equalities

Up until about ten years ago, there was a well-rehearsed argument that advanced industrial societies were fast approaching a digital divide, of users and non-users of the Internet (e.g., Wang et al., 2011). In particular the lower use of digital technologies by older people was described as a “gray digital divide” (Millward, 2003). This was explained by data suggesting that people aged 65 and over had spent the majority of their lives without the Internet and digital technologies. However, the rapid spread of the smartphone across societies has significantly changed this picture. Instead, a majority of the older population now uses smartphones and is thus an active part of the digital world (Pew Research Center, 2021; VuMA, 2021). At the same time, international comparative studies show that the smartphone has inscribed itself deeply into the lifeworlds of older people and has become a central technology for communication, for achieving diverse meaningful goals and a technology with high emotional significance (Miller et al., 2021).

Looking back at the developments of the past ten years, older people have been the fastest growing segment of Internet users compared to younger age groups. Moreover, older users appear to be using digital technologies in similar ways to younger age groups, albeit with a stronger focus on communicating with others (in and outside of social networking sites) and finding information rather than e.g., using financial services (Rosales and Fernández-Ardèvol, 2019; Choudrie et al., 2020). Nonetheless, a conceptual difference is often made between older users who have learnt to adopt digital technologies and keep up with the ever-changing technologies, and cohorts of young people born since 2000, who have been exposed to the digital world from an early age and are therefore referred to as “digital natives”.

Data from Germany from 2003 show that the majority of people aged 60 to 75 today were already using both computers and the internet at that time (Kahle et al., 2004). It can therefore be assumed that the younger cohorts of older people have at least a decade of experience in using digital technologies. This hypothesis is confirmed by recent surveys of older people and information and communication technology (ICT) usage in Germany (Doh, 2020).

Despite the developments along the social category of age, gender and education remain strong determinants of digital use and non-use across age groups. When considering gender, women are more likely to be non-users than men, particularly in older age groups (Doh, 2020). In an exploration of Eurostat data, Doh (2020) shows that for the age group 65–74 years, the difference has decreased from 11% in 2011 to 4% in 2018. In addition, the difference between men and women using the internet is less prominent in countries with high Internet connectivity (e.g., Island, Norway, and Finland) than countries with low Internet connectivity (e.g., Turkey 15%, Greece 11%, and Serbia 10%) (Doh, 2020). Unlike gender, level of formal education remains a stronger predictor of Internet use across 28 European countries for the age group 55–74 years (Doh, 2020). Using the International Standard Classification of Education (ISCED) the difference between high (score 5–7) and low (score 0–2) educational attainment and internet use was 47%. Education is also relevant in younger age groups, e.g., 25–54 years, where the difference between high and low educational attainment in terms of Internet use was 20% (Doh, 2020). With Internet connectivity increasing across countries the assumption is that digital divides will continue to decrease as the trends on age and gender indicate. However, a different picture emerges, when considering digital inequalities in terms of the technologies that drive increasing digitalisation.

With regard to the aim of developing an autonomous and emancipated use of digital technologies the educational background, especially for older adults is still a limiting factor. Individual learning biographies and competencies shape individuals willingness to engage in livelong learning. Especially in institutional settings (Schmidt-Herta and Formosa, 2014).

Post-digital societies have been characterized above all by the widespread use of artificial intelligence (AI) algorithms embedded in digital devices. AI is mainly based on statistical analyses of large quantities of data and the capacities of advanced computational machines to perform fast calculations and find correlations between a high quantity of independent variables and a specific outcome variable. These complex correlations e.g., in form of artificial neural networks are then used for predictions e.g., in the correct classification of a picture. A prerequisite for these supervised learning models are fully labeled datasets^1^. In the process of labeling data the risk increases that persons and behavior is labeled in an unreflected way reproducing existing stereotypical traits or an inappropriate simplification (e.g., man/women, old/young, healthy/unhealthy) (Orwat, 2020). Furthermore, these AI models categorize users with the purpose of an “individualized” user experience (e.g., advertisement, social media content, search results). This feedback represents a subjectification of the user (e.g., by self-assessment against the feedback provided) reproducing social roles and attributions (Reckwitz, 2020).

People regularly use AI when searching online, using social media or communicating verbally with so-called smart speakers. AI systems are now also regularly used to make decisions with regard to credit and insurance policies and even law enforcement agencies, that it raises questions for all users of digital systems about the influence on their own behavior and their own ideas and values. However, this also raises questions around individual and social opportunities for co-creating an algorithmised society.

## Liquid/ambiguous boundaries of private and social spheres

Next to new and persisting social inequalities across all age groups, the post-digital age has contributed to reshaping the boundary between public and private spheres making them more fluid and ambiguous and thus raising questions about how to square modern perceptions of personal integrity with digital-age demands of commercial markets for statistical analyses of mass data (Betancourt, 2018). Following Deleuze (1992), the coding of individuals and society has changed perceptions of dualities in societies: “We no longer find ourselves dealing with the mass/individual pair. Individuals have become “*dividuals*”, and masses, samples, data, markets or “*banks”*” (1992, p. 5, emphasis in original).

Technologies using AI algorithms contribute significantly to the dissolution of privacy and the public sphere (Hagendorff, 2018). The smartphone and wearables are based on a highly individualized technology that collects data about the user *via* a variety of sensors and apps and makes them available for statistical analyses to digital corporations with or without the explicit consent of users. This data is then analyzed by categorizing AI algorithms and reporting them back to the user in the form of subjectifying answers, e.g., by displaying specific advertisements. In this way, the public sphere directly interferes with the user's private sphere *via* digital devices and platforms (Wolf, 2021). This can be seen as an extension of Habermas' theory of the colonization of the lifeworld (Habermas, 1981). This means that the boundary between privacy and the public sphere is dissolved to the extent that the public sphere gains greater insight into the private sphere of users and uses this information to open up new spaces for shaping the everyday lives of users. This is in line with the current argument of Zuboff (2019) that the core of a new surveillance capitalism is the global trade with tracked user data. These user data (e.g., log files of smartphone usage) are a new form of commodity as they have proven to be able to predict and influence human everyday activities and decision making. A second important component of the dissolution of privacy and the public sphere is the so-called context collapse. In addition to the collection and analysis of data by public stakeholders, users themselves overcome the boundaries of privacy by, for example, writing instant messages or posting private content on social media platforms. Through these channels, private and context-specific information enters a space that is ultimately no longer controllable for the user. For example, a picture posted in a family chat can be forwarded by all members of the chat at any time and thus be published in any amount of new social contexts (Davis and Jurgenson, 2014).

In the post-digital age, individuals are in a constant orbit of networks regardless of whether they are on- or offline. They become (in)voluntary providers of information and recipients of personally targeted and tailored messages. As Zuboff (2019) has argued personal information that previously had no economic worth becomes a commodity not of individuals but of corporate markets that trade with them without the data-provider being recompensed, asked for permission, or having consented. Despite these radical changes, the post-digital economy builds on the capitalist system not only in terms of commodification, production, and growth but also in continuous alienation of participants in the market. By merely providing information and not knowing what categories and classifications are developed at a meta level, individuals are deprived of understanding how their personal information is used for economic purposes at large but also in terms of how it is fed back to them individually as recommendations or indeed limitations for their future behavior. This process is not restricted to consumption but also shapes how people access information, and has extended, depending on national regulation, into the political realm thus shaping democratic systems (Staab, 2019).

This raises profound questions as to how individuals might have lost influence and agency over the digital technologies that have come to define both social and private spheres and that surround them at all times. To find answers it is worth going back in time to earlier theorizing of what constitutes the social and what individuals need to master to maintain agency in post-digital societies.

## Adorno's maxim of emancipation toward autonomy

Adorno's radio lectures “Erziehung nach Auschwitz” in 1966 (Adorno, 1966a/2013) provide the model for a type of education that is orientated toward enabling individuals, educators as well as society at large to face difficult facts about modern social life in constructive ways, including recognizing and resisting right-wing populism and extremism, in an age that imposes increasing uncertainty and challenges on individuals. Although Adorno's arguments are centered around the imperative of preventing Auschwitz from happening again (Adorno, 1966a/2013) his conclusions about how to resist and counteract its resurgence are relevant today not only because right-wing populism and oppression is present in many parts of the world but because his critical social theory seeks to understand oppressive structures and change the power relations that constitute them. Thus, for Adorno, only an emancipated society can prevent the reoccurrence of Auschwitz (Adorno, 1966a/2013).

The foundation of Adorno's “*Erziehung nach Auschwitz”* and education to emancipation (*Mündigkeit*) is the analysis and uncovering of mechanisms that produce the uncivilized (the barbaric) in the civilized (Adorno, 1966a/2013, p. 88). Adorno emphasizes the continuity of societal conditions that led to Auschwitz and that not only provide the possibility of a reoccurrence but also restrict the possibilities of preventing a reoccurrence. For this reason, Adorno focuses on the individual and its education to *emancipation and autonomy*, albeit cognizant of limits to this approach (Adorno, 1966a/2013). Central to his pedagogy is not a forming of individuals but a forming of conscious reflection of societal mechanisms that produce the uncivil, the barbaric. In his understanding, education aims to produce resistance to dominant societal principles of reality. Thus *emancipation toward autonomy* becomes the aim of education. Adorno's use of emancipation follows the tradition of Kant's enlightenment theory, in which enlightenment allows humans to lift themselves from their self-incurred immaturity by using their reason, intellect, and wisdom without the guidance of others. According to Adorno, individuals' self-incurred immaturity is systematically (re-)produced through the identification with social norms and role models. Such enforced conformity leads to incongruences between expected roles and the self, which in turn individuals overcome by exaggerating the expected role. Individuals thus become reproducers of these roles and their own immaturity (Adorno, 1966c/2013). To be cognizant of these processes relies on the ability to see and know about social mechanisms of power. *Emancipation toward autonomy* is thus reached through an examination of reality in which a critique and resistance can unfold. Adorno is aware that every examination of reality is bound by some form of conformity to it, but argues this to be a necessity, as emancipation is defined by its point of departure (Adorno, 1966b/2013, p. 107).

## Emancipation to autonomy in a post-digital age

Emancipation to autonomy has gained in acuteness through digitally enhanced globalization. Although this tech economy was not around at the time of Adorno's writing, the underlying economic systems of capitalism that remain relevant to this day, are central to his theorizing. In his critical theory, they obscure class differences of wealth and power behind a façade of leveled democracy (Gartman, 2012). Drawing on Adorno's analysis of culture and inequality, Gartman notes: “The rise of monopoly capitalism concentrates power into fewer and fewer hands, thus intensifying the alienation of work and depriving people of their needs for freedom, individuality, and sociality” (Gartman, 2012, p. 44). This process is accompanied by mass consumer markets with the sole interest of giving consumers immediate satisfaction through sensual pleasure as well as a desire for more. It has been accelerated by everyday digital products and their capacity to evaluate, react to and stimulate their users every action, thus conditioning these in turn. This is particularly relevant in regard to the use of social media platforms such as TikTok or Instagram, leading to the question to what extent the collected user data in combination with corresponding AI algorithms can provoke an involuntary increase in the use of these platforms (Smith, 2021). Indeed, this reflects the logic of digital capitalism, whose aim is primarily to ensure the permanent consumption of platform-inherent content. This is done by behavior-manipulating power that consumers do not notice negatively during use (Epstein and Robertson, 2015; Danaher, 2016; Helbing et al., 2019; Staab, 2019; Zuboff, 2019).

In his critique of mass culture, Adorno (1970/1992) argues that the immediate gratification of consumers through sensual pleasures (here we equate digital devices and online worlds with mass culture), contributes to maintaining economic inequality by providing individuals with a superficial satisfaction of social recognition that prevents them from seeing how they are lulled into passivity and given a reduced view of the world they live in. In doing so, Adorno argues, it prevents individuals from taking action to create a more just and equal society. In this age of ever decreasing individual autonomy and increasing monopolized economic dominance, it would seem that Adorno's maxim of *emancipation to autonomy* is both at risk but also acutely called for.

Adorno aimed his work at children and young people, however, as we have argued elsewhere (Leontowitsch and Wolf, 2019), his thesis can be translated to later life as all ages continue to experience moments of incongruence. These are potent in phases of transition (e.g., retirement, bereavement, and alienation) when a loss of social status is experienced or enforced, but which includes the potential of becoming aware of the underlying societal mechanisms that make the situation acute. Against this understanding it seems possible to translate an education of *emancipation toward autonomy* into later life, with the aim of critically examining normative images of aging that focus on the merits of consumption and productivity (ibid.). Harry Moody is a prominent thinker in developing images or models of aging that strive toward Adorno's maxim: “A critical gerontology must also offer a positive ideal of human development: that is, aging as movement toward freedom beyond domination (autonomy, wisdom, transcendence)” (Moody, 1988; p. 33). Following Moody it is necessary to render visible the hidden interests and contradictions masked by seemingly harmonious ideas of successful and productive aging, and to ask what diverse actions later life can hold. In this process it is important to distinguish between ascribed ideals of later life and what individuals feel and understand to be meaningful to them (Moody and Sasser, 2017). Thus it would appear that people of different ages are equally affected by incongruences and a search for ontological security in a post-digital world that offers a myriad of possibilities and threats and that does not necessarily foster autonomy, wisdom, or transcendence per se. In a practical application of Adorno's critical theory, his pedagogy highlights the importance of enabling individuals to recognize the normality of proliferating experiences of incongruence, and to respond to such experiences by adopting a productive rather than defeatist stance with regard to the increasing complexity and the intensifying contradictions of modern societies in the 21st century (Dahms, 2020). Moreover, the experiences of incongruence is obscured by the use of everyday digital technologies that suggest enhanced autonomy and agency at a superficial level (e.g., ease of navigation places, independent living or making decisions). However, few individuals have a deep understanding of how these technologies (both hardware and software) work or know how to construct them. With large tech companies promoting user engagement at the level of programming and software development, but with the aim of improving their own product lines, individual agency is constrained to choosing from a large variety of pre-chosen digital products. This perpetuates economic services rather than contributing to social equality or solving social problems.

Adorno does not offer practical prescriptions for creating the kind of critical being that he believes modern societies need and deserve. However, his plea for critique, autonomy, and endurance of dissent calls for learning environments, in which individuals are allowed to develop the tools needed in the process of becoming emancipated. In addition, the core elements of Adorno's concept of emancipation (Mündigkeit), such as critical reflection, can also be found in an educational approach to media literacy. Promoting learning processes leads to the development of necessary orientation knowledge and practical knowledge, but also to reflection on one's own media practices and structural aspects of digitalisation (Hugger, 2020). All three components are therefore necessary prerequisites for participation and shaping one's own digital everyday life across all age groups. In the next part of this paper we wish to provide arguments as to why intergenerational learning may be such an environment.

## Enhancing emancipation toward autonomy through intergenerational learning in a post-digital age

In order to talk about intergenerational learning, it is necessary to define what is meant by “generation” and “learning” (Schmidt-Herta, 2014). Generation can be defined by birth cohort or fixed time spans. However, such approaches do not account for or explain why generational groups vary in terms of cohesion, identity or cultural practices. Thus we take as a departing point the constructivist formulation of *generational location* or *generational unit* by Mannheim (1928), in which he analyses generation as certain cohorts molded through the experience of critical historical events during a certain phase of their lives (mainly youth). Mannheim elaborates the concept by a further element, namely participation in a *common destiny* (inneres Ziel) (Mannheim, 1928), which involves active participation rather than just experience, creating a distinct generational *entelechy* (Entelechie) or *style* (Ausdruck) (Mannheim, 1928). In order to conceptualize generational units and their longevity into later life, Gilleard and Higgs (2011) have combined the concept of generation by Mannheim with Brourdieu's concepts “cultural field” and “habitus” (Gilleard and Higgs, 2011, p. 35). Following Bourdieu, a cultural field has an underlying logic that establishes and develops it. As a concept, a cultural field has as it focal point “the range of possible practices that can be realized within it and focuses upon the position of the players within it, rather than the identities of players themselves” (Gilleard and Higgs, 2011, p. 35). In addition, habitus “refers to mostly unconscious practices and forms of experience that arise from and help shape the cultural fields in which they are co-assembled” (Gilleard and Higgs, 2011, p. 36). Looking at present day generational units in the population 60+ show that practices developed in a cultural field continue and are enacted long after their socio-historical point/period of origin. Thus the willingness to try out and use digital technologies by current *generational units* over the age of 70, is linked to *practices* in *cultural fields* from their youth when they rejected notions of “old”, developed a youth culture that was open to trying out “the new” (Gilleard and Higgs, 2011) and partook in an expanding education system that led to jobs that would eventually become digitalised.

Applying the generation unit / cultural field to current cohorts of young people is more difficult as they have lived a shorter amount of time and the impact of social events can not always be apprehended at the moment they take place. Rather the significance of socio-historical events becomes apparent in retrospect. However, as we have argued above, current cohorts of young people belong to the first who have lived with mobile digital technologies since earliest childhood (both as child-users or onlookers), thus providing practices and a cultural field in which the development of a generational unit can take place.

When the term learning is used in the following, it refers to a broad educational science definition of learning that does not subordinate itself to a learning theory but takes a look at the shapes that learning can take. From an educational science perspective, learning can be divided into knowledge-learning, skill-learning, life-learning and learning-learning (Göhlich and Zirfas, 2007). These four forms of learning are strongly intertwined and influence each other. Thus, learning not only results in the acquisition of skills or knowledge, but also has a strong connection to the lifeworld of individuals, and also includes self-initiated strategies making learning part of lifeworld coping processes. Furthermore, all learning processes always represent individual transformation processes that can influence and change an individual's views of the world and their role within it (Göhlich and Zirfas, 2007). Following this conceptualization of learning, learning processes can also be the starting point for critical reflection and the development of empowerment (Mündigkeit).

The learning part in intergenerational learning implies that at least two distinct age groups come together, spend a fixed amount of time together, and establish a new knowledge base or acquire practical skills. The spectrum on which intergenerational learning takes place is wide, ranging from learning from each other; learning together; and learning about one another (Franz and Scheunenpflug, 2009). In terms of coming together, intergenerational learning can take place in families, schools, social networks, community groups, among friends and acquaintances, moving on a spectrum of formal and informal to non-formal learning as well as between on- and offline spaces. Intergenerational learning has been hailed as an important approach to learning (European Commission., 2012) fuelled by the realization that despite growing older populations, family and living arrangement have become fragmented, offering fewer opportunities for young and old to mix and exchange ideas. Such lack of exchange can contribute to a cultural gulf between generations. Thus, intergenerational learning and education is seen to have the potential of overcoming this gap and reaching new forms of solidarity and trust between younger and older generations (Schmidt-Herta and Formosa, 2014). In light of the social inequalities outlined in the first part of this paper, intergenerational learning environments can run the risk of not including participants with less social capital regardless of age. Drawing on Bourdieu, Dean (2017) has argued the need to reflect individual's structurally informed agency and open ways for people to participate in social practices that are outside of their cultural field.

Intergenerational learning is not without further challenges and stereotypical images of aging appear to shape the rationale for much intergenerational learning. Thus, following a seniority principle, older people are seen as “knowledge keepers” who can and should pass their wisdom on to younger generations (Franz and Scheunenpflug, 2009). In terms of digital knowledge, however, older people are seen as having a deficit that can be filled by learning skills from younger so called “digital natives” (e.g., López Seguí et al., 2019). This dichotomy of images runs the risk of engraining age stereotypes and overlooking the heterogeneity of both younger and older generational units as well as the heterogeneity of practices developed and maintained out of cultural fields. At the same time, research suggests that there are qualitative differences between the desires of younger and older learners: older people appear to seek intergenerational setups as a way of having contact with younger people (Schmidt and Tippelt, 2009), whereas younger people are more concerned about understanding the world around them and shaping their identity (Kessler, 2005). How does one bridge these different vantage points? The aim of this paper is to examine the potential of intergenerational learning for *emancipation toward autonomy* in post-digital societies. If this is to be achieved, involuntary intergenerational setups (e.g., in families), simple binary learning processes (e.g., young learn from old, old lean from young) or learning by instruction (e.g., knowledge is departed and absorbed) will not suffice. Instead, volition, reciprocity, mutuality on parts of the participants as well as understanding learning as a co-construction (Moon, 2004) need to be central to intergenerational learning, implying that both generational groups learn from one another, do so freely, and use reflection to jointly gain new knowledge.

## The role of reflection in intergenerational learning in post-digital age

Based on their extended research on intergenerational learning, Franz and Scheunenpflug (2009) developed recommendations for educators. These include the need to encourage and support the exchange of knowledge between the generational groups and to avoid one generational group becoming the “teacher” and the other the “pupil”. This, they argue, contributes to avoiding age stereotypes and one group retreating into a passive position. Next, Franz and Scheunenpflug (2009) recommend that the groups are clearly defined, so that educators (trained persons who bring the generational groups together and moderate the sessions) and the participants know about what learning experiences and habits other participants have, as well as where there might be resistance to learning (e.g., negative past experiences of schooling). Acknowledging difference and explicitly working with difference is key to enabling the respective generational groups to develop curiosity, openness and appreciation for one another at the same time as experiencing and experimenting with different opinions and enduring dissent. Franz and Scheunenpflug (2009) argue, that a transparent approach to dealing with heterogeneity allows participants to change perspectives and ask questions they may not feel free to ask persons of other generations close to them, such as family members. Moreover, getting to know the unknown other helps participants to reach a more reflected picture of themselves (Franz and Scheunenpflug, 2009, p. 448). Thus, educators and learners then have to know how to make use of heterogeneity within the learning group and to understand it as a resource rather than a challenge (Franz and Scheunenpflug, 2010). This takes us to a key learning skill, dealing with the incongruence of heterogeneity through reflection.

Moon (2004) differentiates between *surface approaches* as an attempt to memorize mediated facts (e.g., through educators, printed media or other sources) and *deep approaches* in which the learner seeks to understand the meaning of material in relation to previous knowledge through a process of reflection. For Moon (2004), reflection is a conscious cognitive process that links existing knowledge to an analysis of the relationship between current experiences and future action. It is a form of experiential learning that is relatively unmediated, i.e., it is independent of educators and material, although the latter can support reflection in learning settings. Reflection is concerned with the activity of reorganizing already available knowledge and emotional orientations in order to achieve further insights and to inform next steps. Following Moon (2004), reflective learning is particularly apt to ill-structured and unpredictable situations, which is why this method of learning was developed for professions such as nursing and teaching. Intergenerational learning arrangements can also be described as relatively unstructured und unpredictable as each and every intergenerational project includes different generational units, a spectrum of individual learning preferences and backgrounds, as well as different aims. With a deep learning approach, reflection helps participants to take stock of the knowledge they hold about themselves (individually and as belonging to a generational unit) and what they know about the others they are working with. As the participants get to know each other more, they accumulate new knowledge (experiences) that needs integrating into the knowledge base they already posses. Creating an atmosphere in which new knowledge and conflicting knowledge is deemed positive and “safe”, prevents the risk of only adding knowledge that is in line with previous experiences and ignoring or discarding experiences that appear alien. As Moon (2004) argues, reflection trains and becomes the ability of not only acquiring new knowledge, but of interrogating previously taken-for-granted assumptions.

In this way, intergenerational learning that is concerned with making visible differences and working with differences, creates an arrangement or micro-cosmos, in which participants can safely experience incongruences that they have developed through enforced conformity with social norms and role models and that come to the fore in the intergenerational arrangement. Instead of overcoming incongruences by exaggerating expected roles and thus reproducing those roles and their own immaturity, as Adorno (1966c/2013) argues, intergenerational learning offers a way to support a process of recognizing and knowing about social mechanisms that determine how participants see and relate to the “unknown other”.

Returning to the possibilities and threats of living in the post-digital age, we would argue that intergenerational learning that is concerned with difference and learning about otherness through reflection is well suited to allowing young and old people to ask about how and where digital technologies and AI algorithms are present in their current lives, how and why they use them, how much agency they as users have in relation to these technologies, and, ultimately, how they envisage their future with these technologies. Kade (2009) has argued that older people may need to rekindle their access to the world as rapid technological developments, and the inability to follow them, may have contributed to their alienation and disengagement. Although younger generations appear proficient in adapting to these rapid changes, they may, too, lack understanding about the algorithmic and economic structures that drive the post-digital age and that shapes individual agency and access to the world. Providing a space in which different generational units can come together to answer such questions in a structured way, enables an agentic learning occasion that encourage individuals to reflect on their self-world relations.

## Conclusion: What is needed

This paper has asked, how members of different age groups or generations are framed differently when it comes to problematising life in the post-digital age with respect to digital (in)equalities, and changes in the perceived boundaries of private and social spheres. In addition, it asked how a practical application of Adorno's critical theory can highlight the importance of enabling young and old individuals to recognize the normality of proliferating experiences of incongruences and to respond to such experiences by adopting a productive rather than a defeatist stance. We have argued, that the digital age has amplified experiences of incongruence through increasing complexity and intensifying contradictions, at the same time as lulling users into a sense of congruence through personalized and immediate recognition and appraisal. In following Adorno's idea of not forming individuals but of forming conscious reflection of societal mechanisms, intergenerational learning arrangements are theorized as spaces in which incongruences can be reflected, reassessed, and contained rather than avoided by exaggerating expected ideas and roles of the post-digital age. Adorno's maxim of emancipation toward autonomy seeks to understand oppressive structures and change the power relations that constitute them in constructive ways by recognizing and resisting difficult facts about what Adorno called modern and we have referred to as post-digital life.

Next to generating new knowledge about one another, intergenerational learning projects can create spaces for generations to exchange ideas about their digitalised lives and how to jointly develop ways of living with digital technologies in future. As we have argued, later life capsules many different generational units. This means that in intergenerational learning arrangements a relatively distinct generational group of young people can meet an array of generational units of older people. It will be down to the individual projects to decide whether or not it can handle such heterogeneity or whether it wishes to reduce it by narrowing the cohorts sampled. However, an openness for the origin and development of practices and cultural fields within one or several generational units will be key to supporting learning about one another that goes beyond age and generational stereotypes. All of this calls for educators to pilot such projects and for researchers to evaluate the projects while they are underway.

By providing such a intergenerational learning space—the digital classroom—not a space of online learning but of jointly co-producing knowledge about digital technologies and AI algorithms, the superficial satisfactions of everyday digital technologies can be uncovered for what they are: a seemingly naturalized endless cycle of immediate gratification of consumers through sensual pleasures and creation of new consumer desires. The digital classroom can enhance media competence by analyzing context collapse and the colonalisation of lifeworlds through personal experiences across different biographies allowing participants to become critical agents. By jointly emancipating users of different ages with knowledge and skills about how these technologies work it provides them with the ability to take action for their own lives. At a collective level, it holds the potential of creating a more just and equal society.

## Data availability statement

The original contributions presented in the study are included in the article/supplementary material, further inquiries can be directed to the corresponding author.

## Author contributions

The article was developed and written jointly by ML, FW, and FO. All authors contributed to the article and approved the submitted version.

## Funding

The publication of this article was funded by the University Library of Goethe University Frankfurt.

## Conflict of interest

The authors declare that the research was conducted in the absence of any commercial or financial relationships that could be construed as a potential conflict of interest.

## Publisher's note

All claims expressed in this article are solely those of the authors and do not necessarily represent those of their affiliated organizations, or those of the publisher, the editors and the reviewers. Any product that may be evaluated in this article, or claim that may be made by its manufacturer, is not guaranteed or endorsed by the publisher.

## Footnotes

^1^Although there are other forms of machine learning algorithms (e.g., unsupervised learning and reinforcement learning) supervised learning models represent the vast majority of todays used AI algorithms.
